# Identifying the barriers and facilitators to transforming a university hospital into a Major Trauma Centre: a qualitative case study using the Theoretical Domains Framework

**DOI:** 10.1186/1757-7241-23-S2-A10

**Published:** 2015-09-11

**Authors:** Neil Roberts, Fabiana Lorencatto, Joanna Manson, Jan Jansen

**Affiliations:** 1 Barts Centre for Trauma Sciences, Blizard Institute, Queen Mary, University of London, London, UK; 2 Health Services Research and Management, School of Health Sciences, City University London, London, UK; 3 Department of Intensive Care, Aberdeen Royal Infirmary, Aberdeen, UK

## Background

Scotland is currently implementing a trauma network with four Major Trauma Centres (MTCs). Transforming successful teaching hospitals in to MTCs is likely to generate various beliefs amongst staff. The Theoretical Domains Framework (TDF) is a tool to elicit and analyse beliefs [1]. This study applied the TDF to explore barriers/facilitators to MTC establishment.

## Method

Semi-structured interviews were conducted with 10 participants from a single hospital prior to MTC designation including clinicians, nursing and management staff. A topic guide was designed using the TDF. Interview transcripts were analysed following a framework analysis approach and coded according to TDF domains. Themes were analysed for relevance according to prevalence, expressed importance, discordance and underlying evidence base.

## Results

1728 utterances were coded into 98 themes, of which 57 were classified as relevant barriers/facilitators. Themes addressed 6 key areas: Beliefs towards becoming a MTC (*e.g. My optimism/pessimism is conditional upon availability of necessary resources*), resource demands (*e.g. Recruitment is difficult, and may be affected by (not) becoming a MTC*), current capability (*e.g. Motivation for trauma varies between departments/individuals*), knowledge/skill development (*e.g. Maintaining skills is important, as is developing them*), trauma teams and a structured trauma pathway (*e.g. Someone should lead/coordinate care of trauma patients*), and performance improvement processes (*e.g. The organisational culture is (not) supportive and geared towards performance improvement*).

## Conclusions

This study identified a range of barriers and facilitators likely to influence the transition of this hospital into a MTC. Findings highlight a need for clear systems, processes, communication and teamwork, and delineate the complexity of participants’ motivation and optimism/pessimism. This provides a basis for developing targeted interventions to facilitate the implementation process. This is a replicable method of evidence-based service-improvement, which can be applied elsewhere throughout acute care.
